# Bioluminescent murine models of bacterial sepsis and scald wound infections for antimicrobial efficacy testing

**DOI:** 10.1371/journal.pone.0200195

**Published:** 2018-07-16

**Authors:** Abiodun D. Ogunniyi, Zlatko Kopecki, Elizabeth E. Hickey, Manouchehr Khazandi, Emma Peel, Katherine Belov, Alexandra Boileau, Sanjay Garg, Henrietta Venter, Wei Yee Chan, Peter B. Hill, Stephen W. Page, Allison J. Cowin, Darren J. Trott

**Affiliations:** 1 Australian Centre for Antimicrobial Resistance Ecology, School of Animal and Veterinary Sciences, The University of Adelaide, Roseworthy, South Australia, Australia; 2 Future Industries Institute, University of South Australia, Mawson Lakes, South Australia, Australia; 3 School of Life and Environmental Sciences, The University of Sydney, Sydney, New South Wales, Australia; 4 School of Pharmacy and Medical Sciences, Sansom Institute for Health Research, University of South Australia, Adelaide, South Australia, Australia; 5 Luoda Pharma, Caringbah, New South Wales, Australia; 6 Neoculi Pty Ltd, Burwood, Victoria, Australia; Massachusetts General Hospital, UNITED STATES

## Abstract

There are very few articles in the literature describing continuous models of bacterial infections that mimic disease pathogenesis in humans and animals without using separate cohorts of animals at each stage of disease. In this work, we developed bioluminescent mouse models of partial-thickness scald wound infection and sepsis that mimic disease pathogenesis in humans and animals using a recombinant luciferase-expressing *Staphylococcus aureus* strain (Xen29). Two days post-scald wound infection, mice were treated twice daily with a 2% topical mupirocin ointment for 7 days. For sepsis experiments, mice were treated intraperitoneally with 6 mg/kg daptomycin 2 h and 6 h post-infection and time to moribund monitored for 72 h. Consistent bacterial burden data were obtained from individual mice by regular photon intensity quantification on a Xenogen IVIS Lumina XRMS Series III biophotonic imaging system, with concomitant significant reduction in photon intensities in drug-treated mice. Post-mortem histopathological examination of wounds and bacterial counts in blood correlated closely with disease severity and total flux obtained from Xen29. The bioluminescent murine models provide a refinement to existing techniques of multiple bacterial enumeration during disease pathogenesis and promote animal usage reduction. The models also provide an efficient and information-rich platform for preclinical efficacy evaluation of new drug classes for treating acute and chronic human and animal bacterial infections.

## Introduction

Infection of skin wounds and sepsis caused by pathogenic bacteria that are resistant to multiple classes of antimicrobials account for massive morbidity and mortality in humans and animals worldwide [1–3], with coagulase-positive *Staphylococcus* spp. a leading cause [4–7]. The increasing global prevalence and spread of clinically-relevant antibiotic-resistant bacterial pathogens, particularly methicillin-resistant *S*. *aureus* (MRSA) in hospitals, among hospital workers, veterinarians and within the community is of major public health concern and poses significant impact on health-care costs in many countries [2, 8–11]. Therefore, new drug classes are urgently needed to address this problem. However, animal models for detailed investigation of disease progression in a scenario that mimic bacterial infections in animals and humans often involve using separate cohorts of animals at each stage of the disease process. These assays are laborious, time consuming and involve costly microbiology techniques and materials required for harvesting, plating and enumeration of bacteria derived from infected mice, which are sometimes inconsistent from one day to another.

The development of reproducible and reliable animal infection models that will lead to reduction and refinement of animal usage as well as having the potential to reduce labour and material costs is a significant advance, allowing efficient preclinical evaluation of the efficacy of new drug classes for treating acute and chronic bacterial infections in humans and animals. Bioluminescence is a powerful technique that has been used widely as a reporter system in various *in vitro* and *in vivo* studies, including evaluation of acute and chronic bacterial infections and investigation of antibacterial activities of antibiotics and detection of bacterial resistance to antimicrobials [12–17]. As part of our novel antimicrobial discovery program, reliable and consistent animal infection models to assess preclinical efficacy of drugs designed to treat acute sepsis and/or chronic skin wounds, are of paramount importance. Accordingly, we have developed and optimised bioluminescent models of partial-thickness scald wounds and sepsis in mice by infection with a recombinant luciferase-expressing *Staphylococcus aureus* strain (Xen29) [18].

## Materials and methods

### Bacterial strain and growth conditions

For this study, a bioluminescent derivative of *S*. *aureus* ATCC12600 carrying a modified *lux* operon from *Photorhabdus luminescens* (Xen29) [18], purchased from PerkinElmer, was used. The strain was routinely cultured on Horse Blood Agar (HBA) plate supplemented with 200 μg/ml kanamycin and incubated at 37°C for 18 h before being subcultured into Luria Bertaini (LB) broth supplemented with 200 μg/ml kanamycin and grown to *A*_600_ = 0.5 (equivalent to approx. 1.5 × 10^8^ colony-forming units (CFU)/ml). Bacteria at this density were centrifuged, washed twice in phosphate-buffered saline (PBS) and resuspended to the appropriate density for scald wound infections or sepsis experiments.

### Ethics statements

For partial-thickness scald injury experiments, 6- to 8-week-old male BALB/c mice, weighing between 20 g to 22 g, were used. For sepsis experiments, outbred 5 to 6-week-old male CD1 (Swiss) mice (weighing between 25 g to 32 g), were used. Mice had access to food and water ad libitum throughout the experiments. All mice were obtained from the Laboratory Animal Services breeding facility of the University of Adelaide. The Animal Ethics Committee of The University of Adelaide (approval numbers S-2015-150 and S-2015-151) reviewed and approved all animal experiments. The study was conducted in compliance with the Australian Code of Practice for the Care and Use of Animals for Scientific Purposes (8^th^ Edition 2013) and the South Australian Animal Welfare Act 1985.

### Partial-thickness scald injury and infection experiments

A 69 mm^2^, second-degree, partial thickness burn was created on the dorsal skin of mice as described previously [19] and by a modification of the procedure of Bjorn *et al*. [12], as follows: Mice were anaesthetised by intraperitoneal (IP) administration of xylazine (10 μg/g; Ilium), ketamine (100 μg/g; Ilium) and buprenorphine (0.05 μg/g; Reckitt Benckiser; for pain relief) in 500 μl of 0.9% saline. The dorsum of each mouse was shaved and then placed into a water tight container with the exposed dorsal skin sealed against an aperture using a rubber grommet to prevent leakage and then partially submerged (with breathing holes above the water) into a 65°C water bath for 45 seconds. This was followed by partially submerging the mouse in a cold running water bath (15°C) for 45 seconds to stop the burning process. This procedure creates a highly reproducible, partial thickness burn characterised by the appearance of a blister, oedema and the absence of tissue damage to the underlying fascia and muscle tissues. To minimise pain and discomfort post-procedure, buprenorphine was administered 12 h and 24 h post-procedure. In addition, 500 μl of 0.9% saline was administered IP to mice that showed weight loss at 24 h post-infection. Digital images of wounds were taken daily for macroscopic analysis of burns using optimized protocols [19].

Mice were placed in individually ventilated cages as 3 treatment groups comprising 6 mice per group, as follows: (i) wounded but not infected; (ii) wounded and infected at day 2 post-wounding with bioluminescent *S*. *aureus* Xen29 (1 × 10^7^ CFU in 10 μl PBS) but not treated, or (iii) wounded and infected at day 2 post-wounding with Xen29 (1 × 10^7^ CFU in 10 μl PBS) and then treated from 24 h post-infection, as described below). All mice were imaged immediately after infection on a Xenogen IVIS Lumina XRMS Series III live animal biophotonic imaging system (Caliper Life Sciences). Signals were collected from a defined region of interest (ROI) using the contour ROI tool and total flux intensities (photons/s) analyzed using Living Image Software 4.5. Starting from 24 h post-infection, one group of infected mice were treated twice daily with a total of 200 μl of a 2% mupirocin (as Bactroban®) ointment (equivalent to 4 μg of mupirocin) for 7 days and all mice subjected to bioluminescence imaging daily to quantify bacterial burden. Digital photographs of infected wounds were taken daily and analyzed for macroscopic healing of wounds using the ImageProPlus program (Media Cybernetics Inc., Bethesda, MD, U.S.A.). Power analysis was performed targeting 20% reduction in bacterial luminescence and wound area which would be considered biologically significant and clinically relevant; a sample size of 6 executes this protocol with 95% power using the statistics package G Power 3.1.7.

At the conclusion of the experiment (8 days after commencement of mupirocin treatment), 100 μl blood was withdrawn from each mouse by submandibular bleeding, mice were humanely killed and wounds were collected. Half of each wound was resuspended in 200 μl PBS, vortexed rigorously 3 times over 10 minutes and serially diluted in PBS to assess bacterial load by plating on LB+kanamycin agar. The remaining half was formalin fixed and processed using routine protocols for use in histological assessment of bacterial load and wound healing using Haematoxylin and Eosin stain. Microscopic wound length was determined following standardized methods of manually measuring the distance between wound margins by drawing below the epidermis or clot between the burn wound margins. Microscopic dermal wound gape was determined by measuring between the dermal wound margins. The percentage of the burn wound that had re-epithelialized was determined by measuring the portions of the wound that were covered with epidermis as a percentage of the entire wound. Blinded measurements of histological slides by two independent assessors were performed. Gram stain of the histological section at end point of the experiment also confirmed presence of *S*. *aureus* in infected healing burn wounds following established protocols.

### Sepsis experiments

In order to obtain the optimal challenge dose for *S*. *aureus* sepsis, four groups of Swiss mice (n = 3 per group were initially challenged IP with approx. 5 × 10^6^ CFU, 1 × 10^7^ CFU, 2.5 × 10^7^ CFU, or 5 × 10^7^ CFU of *S*. *aureus* Xen29 in 200 μl PBS containing 3% porcine gastric mucin type III (Sigma Aldrich; Cat No M1778) over a 12 h period. The mice that received 2.5 × 10^7^ CFU of Xen29 produced consistent and reliable infection over the 12 h period; infection of mice with the lower doses were inconsistent, while mice that received the 5 × 10^7^ CFU dose succumbed to infection rapidly and became moribund within 6 h (data not shown). Therefore, the 2.5 × 10^7^ CFU dose was chosen as the optimal dose for subsequent experiments.

For the sepsis challenge and drug treatment experiments, two groups of Swiss mice (n = 6 mice per group) were challenged IP with approx. 2.5 × 10^7^ CFU of Xen29. At 2 h post-infection, approx. 50 μl of blood was withdrawn from the submandibular plexus of all mice for bacterial enumeration after which they were subjected to bioluminescence imaging in both ventral and dorsal positions on the IVIS Lumina XRMS Series III system. Immediately thereafter, group 1 mice received only the drug vehicle (20% (v/v) DMSO in PEG400) IP, while group 2 received daptomycin (as cubicin) at 6 mg/kg IP prepared in drug vehicle. The clinical conditions of all mice were closely monitored, and at 4 h and 6 h post-infection, approx. 50 μl blood was again withdrawn, followed by bioluminescent imaging. After imaging at 6 h post-infection, a second dose of drug vehicle or daptomycin was administered. Mice were further monitored frequently for signs of distress and those that had become moribund or showed any evidence of distress were humanely euthanized by cervical dislocation. At 10 and 16 h post-infection, living mice were further subjected to bioluminescent imaging. In all experiments, signals were collected from a defined ROI using the contour ROI tool and total flux intensities (photons/s) analyzed using Living Image Software 4.5. Correlation of bioluminescence with bacterial CFU in blood at 2 h, 4 h and 6 h post-infection was assessed by the Spearman rank test using Prism GraphPad 7.0c software. Differences in median survival times (time to moribund) for mice between groups were analyzed by the log-rank (Mantel-Cox) tests. Differences in luminescence signals between groups were compared by multiple *t*-tests.

## Results

**Fig 1:**
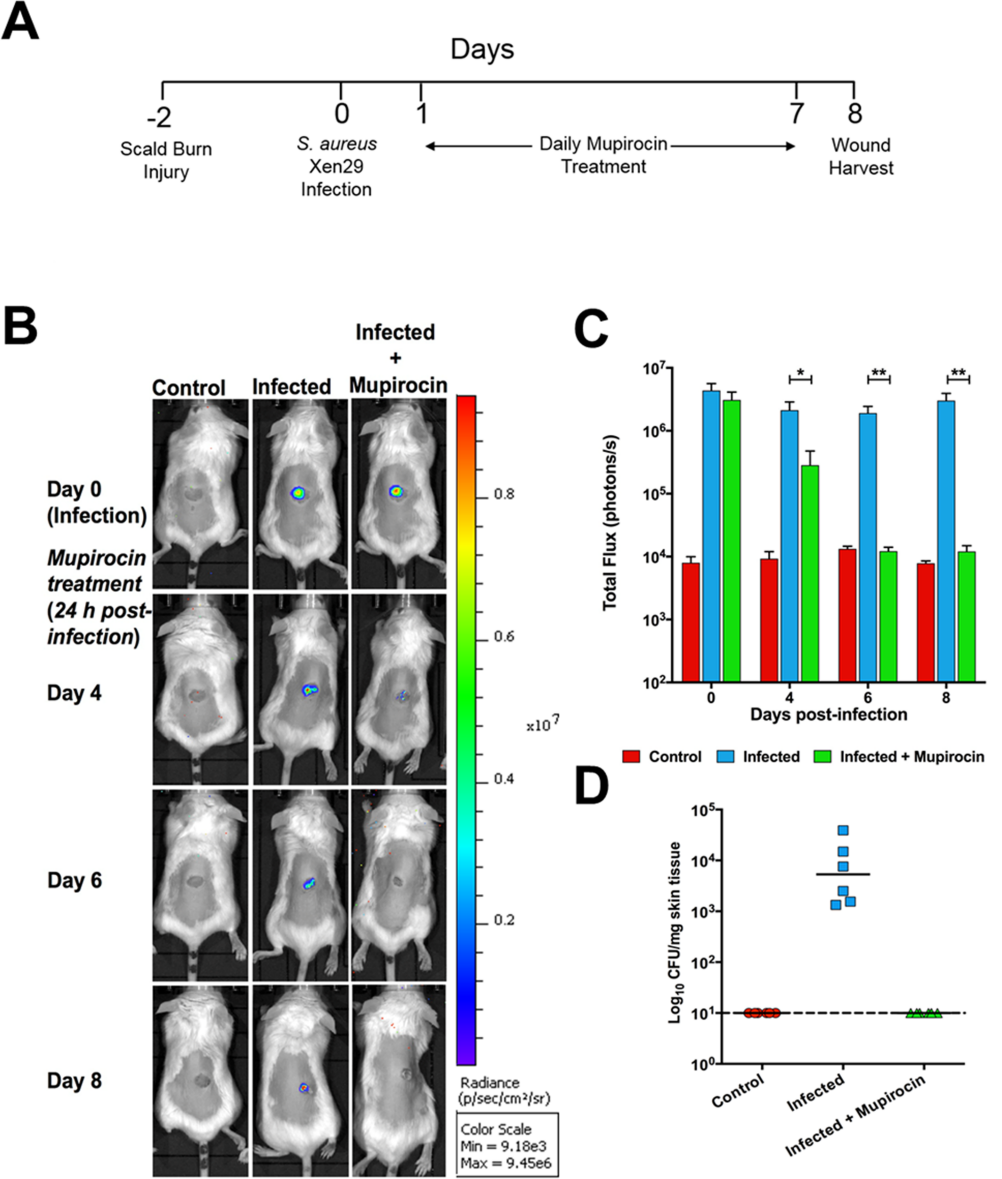
Biophotonic imaging of burn wounds. (**A**) Timeline of scald injury experiments. (**B**) Dorsal images of representative BALB/c mice challenged with approx. 1 × 10^7^ CFU of bioluminescent *S*. *aureus* ATCC 12600 (Xen29). Mice were subjected to bioluminescent imaging on IVIS Lumina XRMS Series III system at the indicated times. (**C**), Quantification of photon intensities of bacterial burden showing significant reduction in photon intensities in mupirocin-treated mice. Total flux (means±SEM photons/s; n = 6 mice). *p<0.05; **p<0.01; multiple *t*-tests). (**D**), Total bacterial counts from tissues of control, infected but not treated, and infected + mupirocin-treated mice at the conclusion of the experiment, showing strong correlation with total photon intensities obtained from each treatment group.----denotes limit of detection; ^_____^ denotes geometric mean counts.

For both partial-thickness scald injury and sepsis experiments, consistent and reproducible bacterial burden data were obtained from individual mice by regular quantification of photon intensity on a Xenogen IVIS Lumina XRMS Series III live animal biophotonic imaging system. The bacterial burden data were also consistent with bacterial viable counts. A timeline representative of the scald injury experiments is shown in **Fig 1A**.

**Fig 2:**
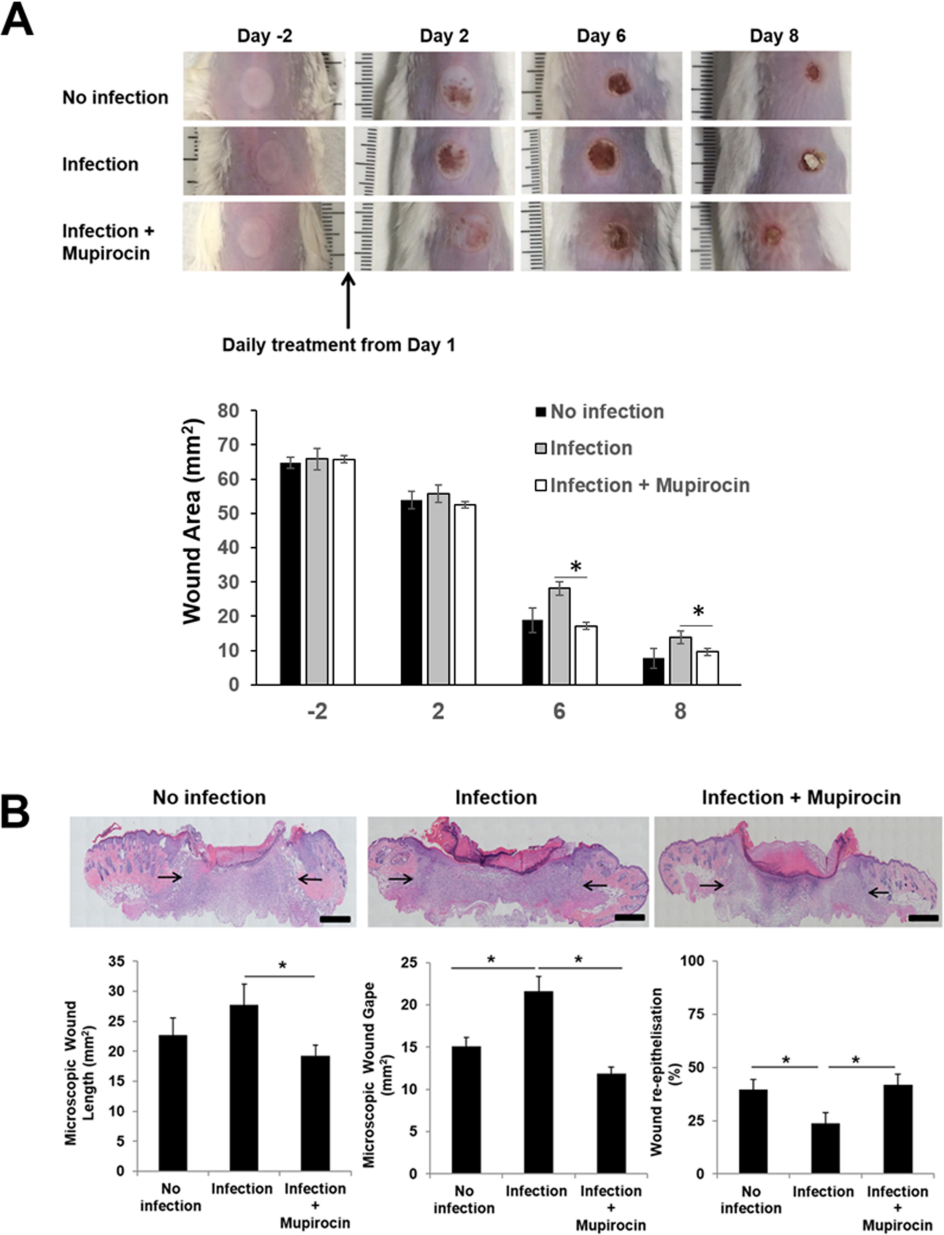
Analysis of wound healing in non-infected, infected but not treated, and infected + mupirocin-treated scald burn wounds. (**A**), Representative images and graphical analysis of scald wounds over a time-course of 10 days illustrating macroscopic differences in rate of healing between non-infected, infected but not treated, and infected + mupirocin-treated mouse scald wounds. (**B**), Representative haematoxylin and eosin-stained sections of partial-thickness scald wounds in mice with non-infected, infected but not treated, and infected + mupirocin-treated wounds. Microscopic analysis of scald wounds at day 10 post-wounding suggests that mupirocin treatment of scald wounds is effective in treating *S*. *aureus* infected wounds leading to significantly decreased wound length, dermal gape and significantly increased wound re-epithelisation compared to infected wounds. Arrows indicate dermal wound gape distance. Magnification ×4 stitched image. Scale bar = 100 μm. Results represent means and SEM, n = 6 wounds per mice group with a single time-point.

For the scald injury experiment, significant reduction in photon intensities in mupirocin-treated mice became apparent 2 days post-treatment (day 4) and reduced to background levels from 4 days post treatment (day 6), which remained until conclusion of the experiment (**Fig 1B and 1C**). Post-mortem analyses showed the bacterial burden in wounds correlated strongly with total flux obtained from bioluminescent signals of Xen29 (**Fig 1D**). Representative digital images of healing wounds are shown in **Fig 2A**, illustrating reproducible wounds at day 0 of the experiment. As a result of scalding the circular wounds developed a white eschar with a surrounding hyperemic zone. Surface wound area was measured in all groups using planimetric analysis of digital images (**Fig 2A**). As expected, the appearance of non-infected burn wounds was clinically superior with decreased inflammation and quicker wound re-epithelization of wounds from day 6 of the experiment. Infection of burn wounds with *S*. *aureus* slowed down the rate of wound healing, as illustrated by larger macroscopic wound area at day 6 of the experiment and presence of the scab at day 8 macroscopically, however no statistical significance was observed in wound length between infected and non-infected burn wounds on microscopic assessment of histological sections (**Fig 2A and 2B**). The main indicators of delayed wound healing in infected burn wounds were significantly increased dermal wound gape and significantly reduced wound re-epithelization compared to both non-infected and infected and mupirocin treated wounds at day 8 of the experiment (**Fig 2A and 2B**). Infected and mupirocin treated wounds appeared paler however more raised at later time-points of the experiment. Macroscopic assessment of mupirocin treated wounds showed significant improvement in healing with smaller wound area macroscopically at both day 6 and 8 of the experiment (**Fig 2A**). In agreement with these findings, histological assessment of these wounds revealed significantly smaller wound length and dermal gape and higher rate of wound re-epithelization compared to infected burn wounds, and appeared similar to the healing profile of non-infected control mice (**Fig 2B**). These findings suggest mupirocin treatment is effective in improving the healing of *S*. *aureus* infected partial thickness scald wounds.

**Fig 3:**
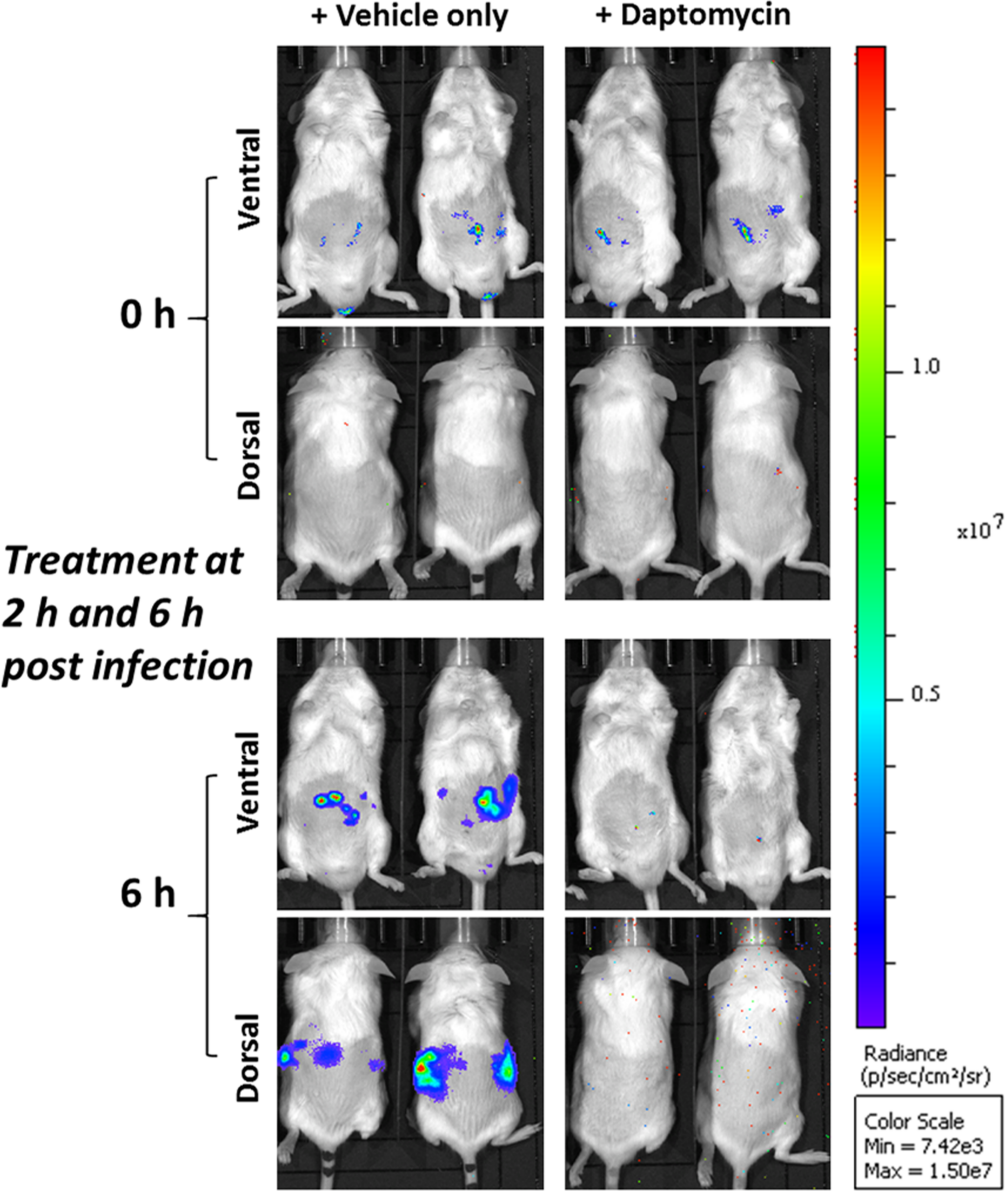
Biophotonic ventral and dorsal images of 2 representative CD1 male mice challenged IP with approx. **2.5 × 10^7^ CFU of bioluminescent *S*. *aureus* ATCC 12600 (Xen29) and then administered the drug vehicle only or daptomycin IP at 2 and 6 h post-infection**. Mice were subjected to bioluminescent imaging on IVIS Lumina XRMS Series III system at the indicated times.

**Fig 4:**
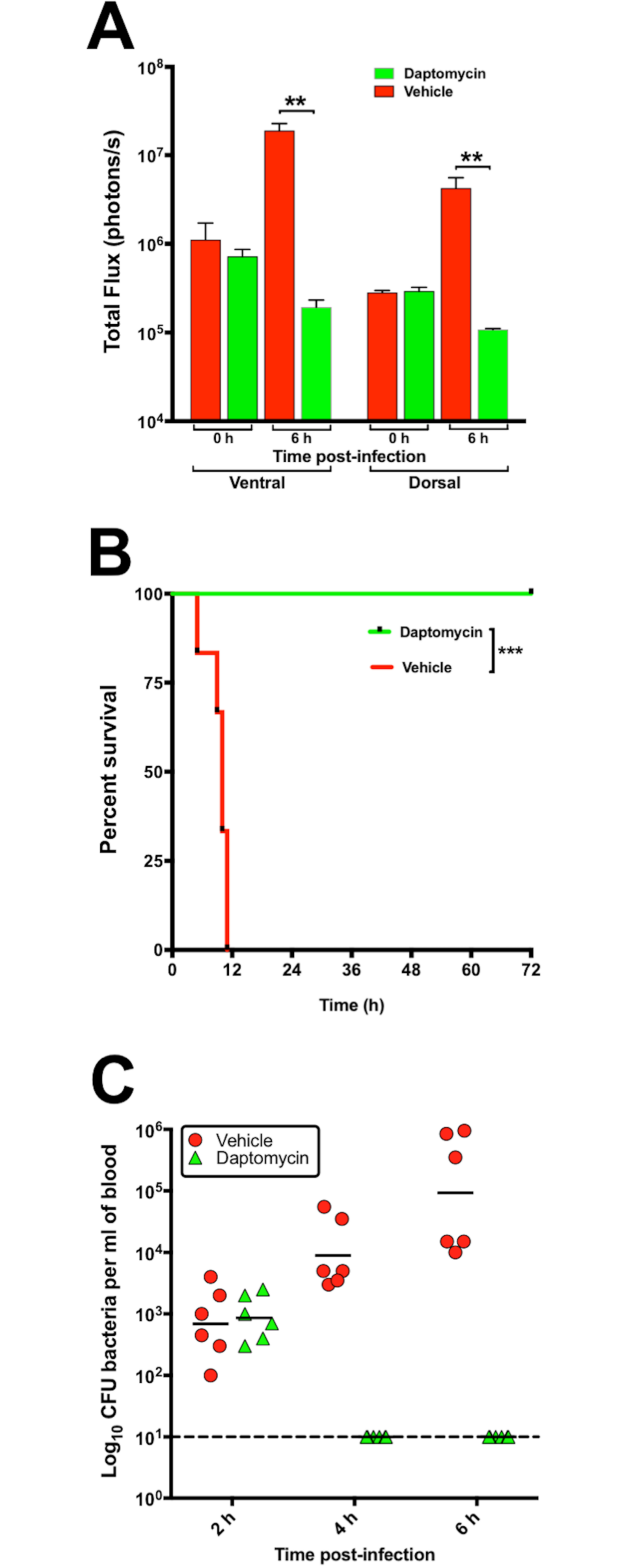
Luminescence signal comparison between groups of CD1 mice challenged IP with *S*. *aureus* ATCC 12600 (Xen29). (**A**), Quantification of photon intensities on a Xenogen IVIS Lumina XRMS Series III live animal biophotonic imaging system, showing significant reduction in photon intensities in daptomycin-treated mice. Total flux (means±SEM photons/s; n = 6 mice). **p<0.01; multiple *t*-tests). (**B**), Survival times for CD1 male mice (n = 6) challenged IP with approx. 2.5 × 10^7^ CFU of bioluminescent *S*. *aureus* Xen29 and administered the drug vehicle only or daptomycin (6 mg/kg) IP at 2 and 6 h post-infection. Differences in median survival times (time to moribund) for mice between groups were analyzed by the log-rank (Mantel-Cox) tests. ***, *P*<0.001. (**C**), Total bacterial counts from blood of control and infected + daptomycin-treated mice at 2, 4 and 6 h post-infection.----denotes limit of detection; ^_____^ denotes geometric mean counts.

The robustness of the bioluminescent model to measure the therapeutic potential of systemically-administered drugs was assessed in an IP sepsis model using *S*. *aureus* Xen29 challenge and subsequent daptomycin treatment, as described in Methods. In these experiments, treatment of mice with 6 mg/kg daptomycin at 2 and 6 h post-Xen29 challenge significantly reduced bacterial burden (**Fig 3**) and total flux (**Fig 4A**). The loss of photon counts correlated with 100% survival of mice (**Fig 4B**) and complete bacterial clearance from blood (**Fig 4C**). Strong correlation of bacterial counts with total photon intensities was obtained at 4 h (*p*<0.05) and 6 h (*p*<0.01) from each treatment group (**S1 Fig**).

## Discussion and conclusions

Bioluminescent models have been used widely to evaluate the efficacy of antimicrobials in treating acute and chronic bacterial infections and to assess bacterial resistance to antimicrobials [13–15, 18, 20–22]. In this study, we have successfully used a well-described *S*. *aureus* strain ATCC12600 carrying a re-engineered *lux* operon from *P*. *luminescens* [18] to establish reliable working mouse models of bacterial scald wound infection and sepsis as robust *in vivo* assays to accurately mimic disease pathogenesis in animals and humans. The two models provide a platform for evaluating the efficacy of new drugs or immunotherapeutic agents against serious bacterial pathogens.

We previously developed the second-degree, partial thickness burn model described here [19]. This model results in approximately 7% of total body surface area burn, and closely represents human second-degree burns in clinical and pathologic aspects as healing is reliant on wound re-epithelization rather than contracture often seen with 3^rd^ degree burn wounds in rodents [12, 23]. To our knowledge, this is the first *in vivo* report of real-time monitoring of *S*. *aureus* infection and treatment after a second-degree, partial thickness burn. The burn model can be reliably applied to evaluate efficacy of therapeutic agents in the healing evolution of deep second-degree burns (in which the dermis remains intact) without the risk associated with a full thickness skin defect.

The use of bioluminescent models to assess pathogenesis of *S*. *aureus* in deeper host tissues and for drug efficacy evaluation is also well documented. These include bioluminescent thigh [13, 24], intravenous [25] and IP [26] models. In this study, we have successfully optimised a similar working model of *S*. *aureus* IP sepsis and kidney infection for efficacy evaluation of new antibiotics in the pipeline of our novel antimicrobial program.

The bioluminescent models described here have several additional advantages over conventional methods, satisfying many aspects of humane animal experimentation. The models promote significant reduction in the number of mice required for long-term studies involving multiple sampling, thereby reducing labour, time and costs associated with harvesting, plating and bacterial enumeration for pathogenesis assessments. Bacterial photon emission from individually-infected mice are consistent and reproducible, and total flux correlates very strongly with total bacterial burden. The fact that a single animal can be sequentially observed for the entire duration of an experiment is an important refinement to existing techniques by eliminating the inherent day-to-day variability in bacterial viable counts associated with random selection and sacrifice of mice. Therefore, the model described here allows for standardization and systematization of techniques and data collection necessary to underpin reliable and reproducible analysis.

There is a potential limitation to using bioluminescence to monitor chronic bacterial infections. Bioluminescence is a product of metabolic activity, as such bacteria that are in stationary phase, such as those in late stage biofilms may not emit sufficient light to establish a correlation with total colony-forming units. In this scenario, a combination of biophotonic imaging and bacterial enumeration at termination of experiments is likely to improve data interpretation.

In conclusion, bioluminescent models of bacterial sepsis and wound infection have been shown to be valuable research tools that can provide a more accurate representation of stages of infection and biofilm formation, thereby promoting better scientific understanding of disease pathogenesis. The models also support preclinical assessment of potential new wound therapies or novel antibacterial agents, and have reduced welfare implications for laboratory animals.

## Supporting information

S1 Fig *S*. *aureus* burden in blood of mice correlates with photon intensity. Correlation of bioluminescence with bacterial CFU counts in blood at 2 h, 4 h and 6 h post-infection was assessed by the Spearman rank test using Prism GraphPad 7.0c software. Positive correlation (r) and statistical significance were obtained at 4 h (*p*<0.05) and 6 h (*p*<0.01) post-infection.
(TIFF)

## References

[1] ECDC. Antimicrobial resistance surveillance in Europe 2014. Annual Report of the European Antimicrobial Resistance Surveillance Network (EARS-Net). Stockholm: ECDC; 2015 2015. 10.2900/23549 PubMed PMID: 26607473.

[2] Groves MD, Crouch B, Coombs GW, Jordan D, Pang S, Barton MD, et al Molecular Epidemiology of Methicillin-Resistant *Staphylococcus aureus* Isolated from Australian Veterinarians. PLoS One. 2016;11(1):e0146034 Epub 2016/01/07. 10.1371/journal.pone.0146034 ; PubMed Central PMCID: PMC4703204. 26735694 PMC4703204

[3] World Health Organization. Antimicrobial resistance: global report on surveillance. 2014.

[4] Fritz SA, Epplin EK, Garbutt J, Storch GA. Skin infection in children colonized with community-associated methicillin-resistant *Staphylococcus aureus*. J Infect. 2009;59(6):394–401. Epub 2009/09/15. 10.1016/j.jinf.2009.09.001 ; PubMed Central PMCID: PMCPMC2788074. 19747505 PMC2788074

[5] Fritz SA, Hogan PG, Camins BC, Ainsworth AJ, Patrick C, Martin MS, et al Mupirocin and chlorhexidine resistance in *Staphylococcus aureus* in patients with community-onset skin and soft tissue infections. Antimicrob Agents Chemother. 2013;57(1):559–68. Epub 2012/11/14. 10.1128/AAC.01633-12 ; PubMed Central PMCID: PMCPMC3535967. 23147738 PMC3535967

[6] Fritz SA, Hogan PG, Hayek G, Eisenstein KA, Rodriguez M, Krauss M, et al *Staphylococcus aureus* colonization in children with community-associated *Staphylococcus aureus* skin infections and their household contacts. Arch Pediatr Adolesc Med. 2012;166(6):551–7. Epub 2012/06/06. 10.1001/archpediatrics.2011.900 ; PubMed Central PMCID: PMCPMC3596005. 22665030 PMC3596005

[7] Muenks CE, Hogan PG, Wang JW, Eisenstein KA, Burnham CA, Fritz SA. Diversity of *Staphylococcus aureus* strains colonizing various niches of the human body. J Infect. 2016;72(6):698–705. Epub 2016/04/06. 10.1016/j.jinf.2016.03.015 ; PubMed Central PMCID: PMCPMC4875821. 27045982 PMC4875821

[8] Hart J, Christiansen KJ, Lee R, Heath CH, Coombs GW, Robinson JO. Increased EMRSA-15 health-care worker colonization demonstrated in retrospective review of EMRSA hospital outbreaks. Antimicrob Resist Infect Control. 2014;3(1):7 Epub 2014/03/05. 10.1186/2047-2994-3-7 ; PubMed Central PMCID: PMCPMC3944736. 24588849 PMC3944736

[9] Knox J, Van Rijen M, Uhlemann AC, Miller M, Hafer C, Vavagiakis P, et al Community-associated methicillin-resistant *Staphylococcus aureus* transmission in households of infected cases: a pooled analysis of primary data from three studies across international settings. Epidemiol Infect. 2015;143(2):354–65. Epub 2014/04/26. 10.1017/S0950268814000983 ; PubMed Central PMCID: PMCPMC4981654. 24763185 PMC4981654

[10] Turnidge JD, Kotsanas D, Munckhof W, Roberts S, Bennett CM, Nimmo GR, et al *Staphylococcus aureus* bacteraemia: a major cause of mortality in Australia and New Zealand. Med J Aust. 2009;191(7):368–73. Epub 2009/10/08. . 19807625 10.5694/j.1326-5377.2009.tb02841.x

[11] Thampi N, Showler A, Burry L, Bai AD, Steinberg M, Ricciuto DR, et al Multicenter study of health care cost of patients admitted to hospital with *Staphylococcus aureus* bacteremia: Impact of length of stay and intensity of care. Am J Infect Control. 2015;43(7):739–44. Epub 2015/03/15. 10.1016/j.ajic.2015.01.031 . 25769617

[12] Bjorn C, Noppa L, Naslund Salomonsson E, Johansson AL, Nilsson E, Mahlapuu M, et al Efficacy and safety profile of the novel antimicrobial peptide PXL150 in a mouse model of infected burn wounds. Int J Antimicrob Agents. 2015;45(5):519–24. Epub 2015/02/05. 10.1016/j.ijantimicag.2014.12.015 . 25649371

[13] Francis KP, Joh D, Bellinger-Kawahara C, Hawkinson MJ, Purchio TF, Contag PR. Monitoring bioluminescent *Staphylococcus aureus* infections in living mice using a novel *lux*ABCDE construct. Infect Immun. 2000;68(6):3594–600. ; PubMed Central PMCID: PMC97648. 10816517 10.1128/iai.68.6.3594-3600.2000 PMC97648

[14] Francis KP, Yu J, Bellinger-Kawahara C, Joh D, Hawkinson MJ, Xiao G, et al Visualizing pneumococcal infections in the lungs of live mice using bioluminescent *Streptococcus pneumoniae* transformed with a novel gram-positive lux transposon. Infect Immun. 2001;69(5):3350–8. Epub 2001/04/09. 10.1128/IAI.69.5.3350-3358.2001 ; PubMed Central PMCID: PMCPMC98294. 11292758 PMC98294

[15] Henken S, Bohling J, Martens-Lobenhoffer J, Paton JC, Ogunniyi AD, Briles DE, et al Efficacy profiles of daptomycin for treatment of invasive and noninvasive pulmonary infections with *Streptococcus pneumoniae*. Antimicrob Agents Chemother. 2010;54(2):707–17. Epub 2009/11/18. 10.1128/AAC.00943-09 ; PubMed Central PMCID: PMCPMC2812129. 19917756 PMC2812129

[16] Orihuela CJ, Gao G, McGee M, Yu J, Francis KP, Tuomanen E. Organ-specific models of *Streptococcus pneumoniae* disease. Scand J Infect Dis. 2003;35(9):647–52. Epub 2003/11/19. . 14620149 10.1080/00365540310015854

[17] Parveen A, Smith G, Salisbury V, Nelson SM. Biofilm culture of *Pseudomonas aeruginosa* expressing lux genes as a model to study susceptibility to antimicrobials. FEMS Microbiol Lett. 2001;199(1):115–8. Epub 2001/05/18. . 11356577 10.1111/j.1574-6968.2001.tb10660.x

[18] Kadurugamuwa JL, Sin L, Albert E, Yu J, Francis K, DeBoer M, et al Direct continuous method for monitoring biofilm infection in a mouse model. Infect Immun. 2003;71(2):882–90. Epub 2003/01/24. 10.1128/IAI.71.2.882-890.2003 ; PubMed Central PMCID: PMCPMC145362. 12540570 PMC145362

[19] Adams DH, Ruzehaji N, Strudwick XL, Greenwood JE, Campbell HD, Arkell R, et al Attenuation of Flightless I, an actin-remodelling protein, improves burn injury repair via modulation of transforming growth factor (TGF)-beta1 and TGF-beta3. Br J Dermatol. 2009;161(2):326–36. Epub 2009/06/13. 10.1111/j.1365-2133.2009.09296.x . 19519830

[20] Campbell-Valois FX, Sansonetti PJ. Tracking bacterial pathogens with genetically-encoded reporters. FEBS Lett. 2014;588(15):2428–36. Epub 2014/05/27. 10.1016/j.febslet.2014.05.022 . 24859085

[21] Kadurugamuwa JL, Sin LV, Yu J, Francis KP, Kimura R, Purchio T, et al Rapid direct method for monitoring antibiotics in a mouse model of bacterial biofilm infection. Antimicrob Agents Chemother. 2003;47(10):3130–7. Epub 2003/09/25. 10.1128/AAC.47.10.3130-3137.2003 ; PubMed Central PMCID: PMCPMC201124. 14506020 PMC201124

[22] Pletzer D, Mansour SC, Wuerth K, Rahanjam N, Hancock RE. New Mouse Model for Chronic Infections by Gram-Negative Bacteria Enabling the Study of Anti-Infective Efficacy and Host-Microbe Interactions. MBio. 2017;8(1). Epub 2017/03/02. 10.1128/mBio.00140-17 ; PubMed Central PMCID: PMCPMC5347345. 28246361 PMC5347345

[23] Simonetti O, Lucarini G, Orlando F, Pierpaoli E, Ghiselli R, Provinciali M, et al Role of Daptomycin on Burn Wound Healing in an Animal Methicillin-Resistant *Staphylococcus aureus* Infection Model. Antimicrob Agents Chemother. 2017;61(9). Epub 2017/07/12. 10.1128/aac.00606-17 . 28696234 PMC5571319

[24] Kuklin NA, Pancari GD, Tobery TW, Cope L, Jackson J, Gill C, et al Real-time monitoring of bacterial infection in vivo: development of bioluminescent staphylococcal foreign-body and deep-thigh-wound mouse infection models. Antimicrob Agents Chemother. 2003;47(9):2740–8. Epub 2003/08/26. 10.1128/AAC.47.9.2740-2748.2003 ; PubMed Central PMCID: PMCPMC182637. 12936968 PMC182637

[25] Plaut RD, Mocca CP, Prabhakara R, Merkel TJ, Stibitz S. Stably luminescent *Staphylococcus aureus* clinical strains for use in bioluminescent imaging. PLoS One. 2013;8(3):e59232 Epub 2013/04/05. 10.1371/journal.pone.0059232 ; PubMed Central PMCID: PMCPMC3595258. 23555002 PMC3595258

[26] Mortin LI, Li T, Van Praagh AD, Zhang S, Zhang XX, Alder JD. Rapid bactericidal activity of daptomycin against methicillin-resistant and methicillin-susceptible *Staphylococcus aureus* peritonitis in mice as measured with bioluminescent bacteria. Antimicrob Agents Chemother. 2007;51(5):1787–94. Epub 2007/02/20. 10.1128/AAC.00738-06 ; PubMed Central PMCID: PMCPMC1855546. 17307984 PMC1855546

