# Supplementary figures and images for: Bioluminescent murine models of bacterial sepsis and scald wound infections for antimicrobial efficacy testing
Source: PLoS One. 2018 Jul 16;13(7):e0200195. doi: 10.1371/journal.pone.0200195 (PMC6047774; doi:10.1371/journal.pone.0200195)

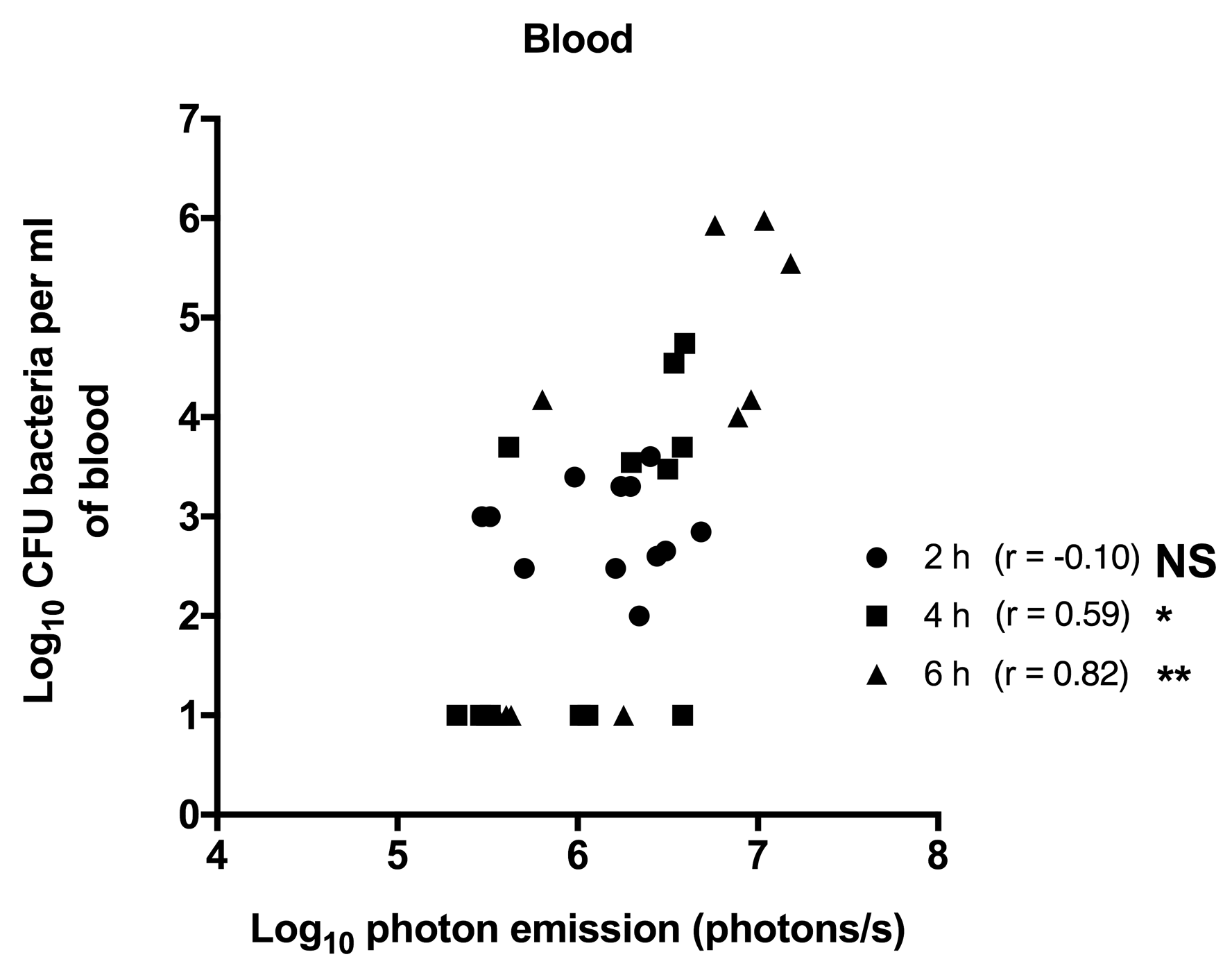

Supplement: S1 Fig — Correlation of bioluminescence with bacterial CFU counts in blood at 2 h, 4 h and 6 h post-infection was assessed by the Spearman rank test using Prism GraphPad 7.0c software. Positive correlation (r) and statistical significance were obtained at 4 h (p<0.05) and 6 h (p<0.01) post-infection. (TIFF) [file pone.0200195.s001.tiff]
